# A deep neural network using audio files for detection of aortic stenosis

**DOI:** 10.1002/clc.23826

**Published:** 2022-04-19

**Authors:** Ingo Voigt, Marc Boeckmann, Oliver Bruder, Alexander Wolf, Thomas Schmitz, Heinrich Wieneke

**Affiliations:** ^1^ Department of Cardiology and Angiology, Contilia Heart and Vascular Center Elisabeth‐Krankenhaus Essen Essen Germany

**Keywords:** aortic stenosis, artificial intelligence, auscultation, deep neural network, machine learning, valvular heart disease

## Abstract

**Background:**

Although aortic stenosis (AS) is the most common valvular heart disease in the western world, many affected patients remain undiagnosed. Auscultation is a readily available screening tool for AS. However, it requires a high level of professional expertise.

**Hypothesis:**

An AI algorithm can detect AS using audio files with the same accuracy as experienced cardiologists.

**Methods:**

A deep neural network (DNN) was trained by preprocessed audio files of 100 patients with AS and 100 controls. The DNN's performance was evaluated with a test data set of 40 patients. The primary outcome measures were sensitivity, specificity, and F1‐score. Results of the DNN were compared with the performance of cardiologists, residents, and medical students.

**Results:**

Eighteen percent of patients without AS and 22% of patients with AS showed an additional moderate or severe mitral regurgitation. The DNN showed a sensitivity of 0.90 (0.81–0.99), a specificity of 1, and an F1‐score of 0.95 (0.89–1.0) for the detection of AS. In comparison, we calculated an F1‐score of 0.94 (0.86–1.0) for cardiologists, 0.88 (0.78–0.98) for residents, and 0.88 (0.78–0.98) for students.

**Conclusions:**

The present study shows that deep learning‐guided auscultation predicts significant AS with similar accuracy as cardiologists. The results of this pilot study suggest that AI‐assisted auscultation may help general practitioners without special cardiology training in daily practice.

## 1 BACKGROUND

After mitral regurgitation, aortic valve stenosis (AS) is the second most common valvular disease.^1^ Its prevalence in the general population is 0.4%, with a sharp age‐dependent increase in the older population. A prevalence of 3.4% in the age cohort >65 years can be observed.^2^ It leads more often to hospitalization than other heart valve diseases and accounts for 45% of patients operated for valvular disease.^3^

It is well known that symptomatic AS has high mortality when no valve replacement therapy is performed.^4^ However, recent data suggest that even asymptomatic severe AS is associated with a mortality of up to 58% within 8 years.^5^ Besides, it has been shown that aortic valve replacement in patients with severe asymptomatic AS significantly improves outcome.^6^ Unfortunately, a high number of patients with significant AS remains undetected. The OxVALVE Population Cohort Study shows that unrecognized, significant AS was present in 1.6% of patients ≥65 years.^7^

Thus, a reliable, readily available, and cheap screening tool is necessary. Since the invention of the stethoscope by Laennec in 1816, cardiac auscultation has been one of the pillars of cardiovascular examination.^8^ However, this method's significant drawbacks are that heart murmurs are variable, and auscultation skills are highly performer‐dependent.^9^

Deep learning is a branch of machine learning using artificial neural networks that models human brains' architecture. Heart sound classification can be principally done using a convolutional neural network (CNN), a subform of deep neural networks (DNNs).^10^ We hypothesized that we could train a DNN to identify heart murmurs suspicious for significant AS with an accuracy comparable to experienced cardiologists.

## 2 METHODS

The study consists of two parts. In the first part, we trained a neural network to classify auscultation findings of patients who have significant AS or not. In the second part, we compared the performance of the trained DNN with the auscultatory skills of 10 experienced cardiologists, 10 residents, and 10 medical students by using a test data set that consisted of a completely disjointed set of patients.

For training, we used auscultation audio files from 100 patients with significant AS and 100 patients without AS. The ground truth was defined by echocardiography. Significant AS was defined as *V*_max_ of >3.5 m/s measured by continuous‐wave Doppler. Although the definition of high‐grade AS has not yet been reached, we chose this cut‐off value, as these patients require close monitoring. Patients admitted for suspicious coronary artery disease or other cardiac diseases were taken as a control group.

We used an electronic stethoscope (Eko) connected to a smartphone interface via Bluetooth for auscultation. Auscultation was performed at the aortic auscultation point (second intercostal space, right sternal border) and the mitral auscultation point (fifth intercostal space, midclavicular line). Thus, from each patient, we included two auscultation files. At each auscultation point, audio files with an interval of 15 s and a sampling rate of 40 kHz were recorded.

The audio files were recorded as part of the clinical routine in a tertiary teaching hospital with a large valve unit specialized in transcatheter aortic valve implantation (TAVI). Only data from patients of this database were included who got echocardiography within 7 days before or after auscultation. Since the study was retrospective, an explicit ethics vote was not necessary according to the regulations of the responsible ethics committee.

We preprocessed the data in our study before using them to train the network. In the first step, the 15 s sound files were divided into three equal parts of 5 s. This was done to overcome the risk that small portions of an auscultation file falsified by respiration^11^ contribute disproportionately to the training of the whole network. Consequently, six sound files per patient (three files for each of the 2 auscultation points) contribute to the network's training. In the second step, we performed Mel Frequency Cepstral Coefficients transformation of the audio files (MFCC‐transformation). This transformation maps the perception of human hearing and has been proposed for audio data analysis of heart and lung auscultation.^12^, ^13^, ^14^ Subsequently, we trained the DNN with 1200 processed audio files (6 files per patient, 100 patients with AS, 100 patients without AS).

**Figure 1:**
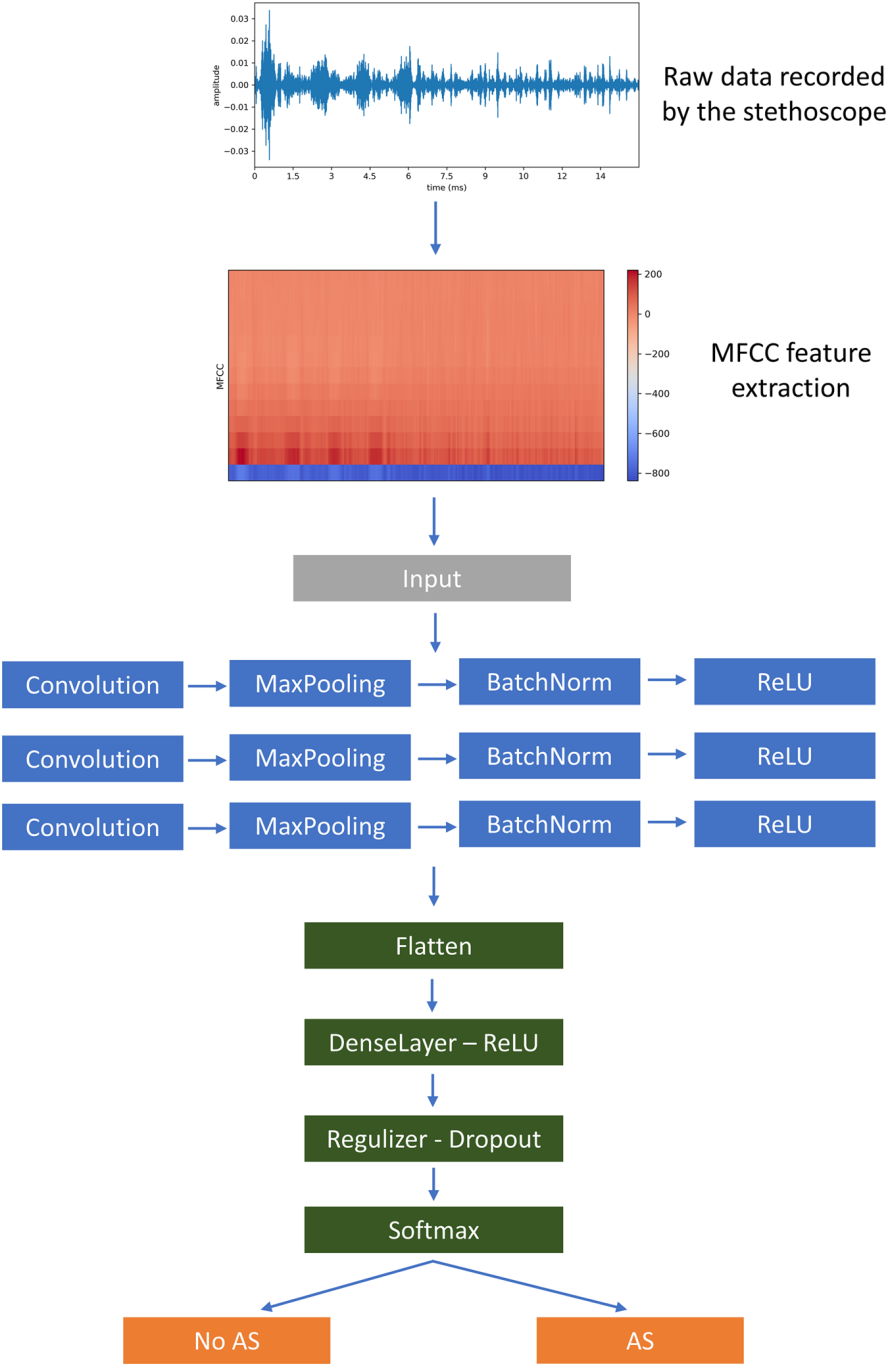
Data processing and analysis were principally done in two steps. In the first step, MFCC feature extraction was done. In the second step, the preprocessed data were fed to the convolutional part of the DNN. After the convolutional layers, the output is flattened to a one‐dimensional tensor. Data are then fed to a fully connected layer using the ReLU (rectified linear unit) activation function. To overcome overfitting, which means that the network is too much adapted to the training data set, the regulizer and dropout techniques were applied. In the softmax function, the input values are transformed to a probability distribution that gives the probability of AS or no AS in the present case. AS, aortic valve stenosis; MFCC, Mel frequency cepstral coefficients.

We developed a CNN for classification (Figure 1), which takes as input MFCCs. The sequential model has three two‐dimensional‐convolutional layers and one max pooling layer. As an activation function, we used “ReLU”. Before each convolutional layer, we applied batch normalization. To combat overfitting, we used a dropout layer between the convolutional layers that sets a random portion of the weights equal to a probability of 0.2 to 0. Thereby the network has to learn different aspects of the data each time.^15^

Hyperparameter tuning was done iteratively for learning rate, batch size, number of epochs, number of kernels, and grid size of the convolutional layers. Model comparison was made using *K*‐fold cross‐validation.

After training the network, it was applied to the test set that the model had not seen before. The test set consists of 20 patients with AS and 20 without AS. Accuracy, sensitivity, specificity, receiver operating characteristic curves (ROC), and F1 score were calculated. F1 score was calculated using the following formula: F1 score = 2 × (recall × precision)/(recall + precision). Then the same test set was classified by 10 experienced cardiologists, 10 residents, and 10 final year medical students. The performance parameters were averaged in cardiologists, residents, and students.

Audio file processing, training the DNN, and making predictions were made with the general‐purpose programming language Python. Preprocessing audio files was done by the audio analysis library Librosa. We generated Mel Frequency Cepstral Coefficients (MFCC) with a hopelength of 10 and 13 coefficients.^16^ A CNN was implemented using the Keras framework with a TensorFlow (Google) backend.^17^

Continuous baseline characteristics are given as mean ± SD. Continuous variables were compared using *t*‐test, and categorical variables were compared using chi‐quadrat test. Accuracy, sensitivity, specificity, and F1‐value for cardiologists, residents, and students are given as mean with a 95% confidence interval. The confidence intervals for the specific parameters of the DNN were calculated with the formula = *z *× sqrt((parameter × (1 − parameter))/*n*), where *z* is the corresponding parameter and *n* is the size of the test sample, 40 in the present case. Inter‐rater reliability was assessed by calculating Fleiss' kappa.^18^

## 3 RESULTS

Data from 120 patients with and 120 patients without AS were taken for the present study. From each group, audio files from 100 patients were allocated to the training group and 20 to the test group, respectively.

Of the 120 patients with AS, in 99 patients femoral TAVI, in 5 transapical aortic valve implantation, in 13 patients open‐heart surgery, and in 1 patient only valvuloplasty were performed. Two patients were treated conservatively.

Patients with AS were older than control patients in the training and test patients. A significant proportion of patients had mitral or tricuspid valve disease. Atrioventricular valve defects were more frequent in patients with AS. However, this difference was not significant. For details on patient characteristics, see Table 1.

**Table 1:** Patient characteristics

	Training set		Test set	
	No AS (*n *= 100)	AS (*n *= 100)	No AS (*n *= 20)	AS (*n *= 20)
Male (%)	60	52	55	50
Age (years)	75.5 ± 10.2	80.5 ± 6.6*	74.7 ± 7.7	80.4 ± 5.6*
BMI (kg/m^2^)	27.5 ± 4.2	27.3 ± 4.2	26.9 ± 4.1	28.4 ± 5.4
Sinus rhythm (%)	86	94	80	95
LV‐ejection fraction (%)	55.0 ± 9.2	55.1 ± 8.0	54.8 ± 8.7	52.6 ± 10.3
Aortic valve regurgitation^a^ (%)	7	16	10	10
Mitral valve regurgitation^a^ (%)	19	21	15	25
Tricuspid valve regurgitation^a^ (%)	13	18	5	20
Aortic valve *V*_max_ (m/s)	1.4 ± 0.3	4.2 ± 0.5**	1.3 ± 0.3	4.3 ± 0.5**
*P*_mean_ (mmHg)	ND	46.2 ± 15.0	ND	47.0 ± 10.1
AVA/BSA (cm^2^/m^2^)	ND	0.8 ± 0.2	ND	0.8 ± 0.2

*Note*: Data are given as mean ± SD.

^a^ Moderate or severe heart valve disease.

*p < .05 no AS versus AS. **p < .01 no AS versus AS.

### 3.1 DNN's diagnostic accuracy

Hyperparameter tuning was done using *K*‐fold cross‐validation. The training data were split into fourfolds, and while iterating through the folds, each iteration uses onefold as the validation set. Using this approach, the optimized DNN consists of three convolutional layers with 32 kernels with a grid size of 3 × 3 (convolutional layer 1 + 2) and 2 × 2 (convolutional layer 3). The best results could be achieved with 40 epochs, a batch size of 4, and a learning rate of 0.001. After flattening the tensor, a fully connected layer with 150 nodes followed. The final fully connected softmax layer produces a distribution over the two output classes. A schematic of the neural network is shown in Figure 1.

We analyzed whether the algorithm showed different results depending on the auscultation point. As expected, worse prediction accuracy is shown when the neural network was trained only with audio files from one auscultation point. This is most likely due to the smaller number of audio files. However, the cause might also be that both auscultation points provide complementary information. Actually, the performance of a model trained only with data from the mitral point was the same as when only trained with data from the aortic point. This result contradicts common clinical experience. However, DNNs are able to identify patterns that are not recognizable to humans. The ROC‐AUC of the DNN trained only with audio files from the aortic auscultation point was 0.93, only trained with audio files from the mitral auscultation point was 0.83 and for merged data was 0.99. The DNN did not detect two patients with AS. No patient without AS was misclassified. The positive predictive value for the DNN using both auscultation points was 1.0, for students 0.83 (0.81–0.86), for residents 0.86 (0.82–0.89), and for cardiologists 0.93 (0.91–0.94). The negative predictive value for the DNN using both auscultation points was 0.91 (0.82–1.0), for students 0.94 (0.93–0.95), for residents 0.94 (0.93–0.95), and for cardiologists 0.96 (0.94–0.97).

### 3.2 Diagnostic accuracy of the CNN versus cardiologists, residents, and students

**Figure 2:**
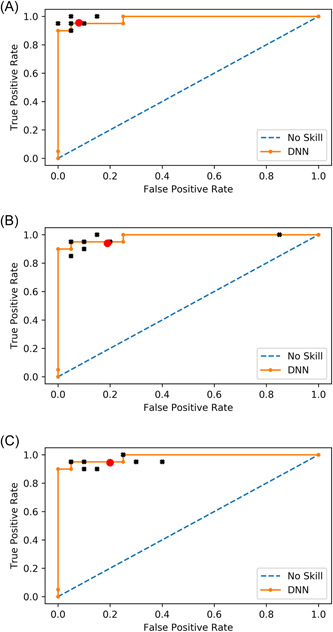
ROC curve (orange line) achieved by the model in comparison to students (A), residents (B), and cardiologists (C). Individual rater performance is indicated by the black crosses, and averaged cardiologist performance is indicated by the red dot.

Ten students, 10 residents in an advanced stage of training, and 10 consultant cardiologists participated in the study. Participants were blinded for the results of the DNN. They were asked to classify patients whether to have AS using two audio files for each patient. For inter‐rater reliability, Fleiss' kappa was 0.69 in students, 0.64 in residents, and 0.84 in cardiologists. This shows that the agreement in the group of cardiologists is much higher than in the group of residents and students. The F1‐score is a parameter to compare the performance of different models or rater groups when seeking a balance between precision and recall. In Figure 2 ROC curves for deep learning model, students, residents, and cardiologists are shown. The DNN showed a higher F1‐score than the mean score of cardiologists, residents, and students. Values for accuracy, sensitivity, specificity, and F1‐score are given in Table 2.

**Table 2:** Comparison between the DNN and humans in the detection of AS in the test set

	Accuracy	Sensitivity	Specificity	F1‐score
Sounds files form aortic position	0.8 (0.68–0.92)	0.8 (0.68–0.92)	0.8 (0.68–0.92)	0.8 (0.68–0.92)
Sound files from mitral position	0.8 (0.67–0.92)	0.9 (0.81–0.99)	0.7 (0.56–0.84)	0.81 (0.70–0.94)
Sound files from both positions	0.95 (0.88–1.0)	0.9 (0.81–0.99)	1.0	0.95 (0.89–1.0)
Human skills				
All participants (*n *= 30)	0.90 (0.88–0.91)	0.95 (0.93–0.96)	0.84 (0.81‐0.87)	0.90 (0.81‐0.99)
Cardiologists (*n *= 10)	0.94 (0.91–0.96)	0.93 (0.91–0.96)	0.96 (0.92–0.98)	0.94 (0.86–1.0)
Residents (*n *= 10)	0.88 (0.84–0.91)	0.94 (0.90–0.97)	0.81 (0.75–0.86)	0.88 (0.78–0.98)
Students (*n *= 10)	0.87 (0.84–0.90)	0.95 (0.90–0.97)	0.80 (0.74–0.85)	0.88 (0.78–0.98)

*Note*: Data are given as mean and confidence intervals.

Abbreviations: DNN, deep neural network; MR, mitral regurgitation.

## 4 DISCUSSION

The present study shows that deep learning‐guided auscultation predicts significant AS with similar accuracy as board‐certified cardiologists. These results suggest that artificial intelligence‐assisted auscultation may help general practitioners without special cardiology training.

Auscultation is one of the pillars of clinical investigation. It is readily available, and no sophisticated technical requirements are necessary. On the other hand, a high level of expertise is essential, and the skills, once acquired, need to be used continuously. These circumstances may explain errors in auscultation between 20% and 80% in residents, primary care physicians, and cardiologists.^19^, ^20^

For this reason, computer‐assisted auscultation was already proposed for clinical use at the beginning of this century. The developed algorithms decompose the cyclical heart sound, and hand‐engineered processing is applied for classification. In young patients with hypertrophic cardiomyopathy and children with congenital heart disease, sufficient sensitivity and specificity could be achieved with these techniques.^21^ However, these studies were done in young patients with no conditions complicating the auscultation results like adiposity and lung emphysema.

Deep neural networks (DNNs) take a completely different direction. A DNN is a machine learning algorithm that models brain architecture. They consist of perceptrons with adjustable weights and activation thresholds. In supervised learning, labeled data, often called ground truth, are propagated through a network of perceptrons that allows the DNN to adjust weights. In this step, the DNN learns how to assign known data to predefined categories. After this training phase, the performance of the final model is evaluated on a test data set. In this step, the trained DNN makes predictions in the form of probabilities for so far unknown data.

In first applications, the focus was directed on standard diagnostic techniques used by doctors daily but cannot be provided on an expert level in any case.^22^ Deep learning systems were developed for ECG interpretation,^23^ skin cancer identification,^24^ and papilledema detection.^25^

In this context, it has been recognized that AI may also be a valuable tool to support doctors in identifying valvular heart disease. In a recently published study by Chorba et al., physicians assigned 5878 auscultation findings to the labels “heart murmur”, “no heart murmur”, or “inadequate signal”. The DNN was trained with these data using an end‐to‐end (E2E) network design. In a second step, the DNN was then validated on a test data set of 1774 recordings annotated by separate expert clinicians.^26^

In contrast, the ground truth was not defined by physician assignment but by echocardiography in the present study. By using this gold standard, the well‐known erroneous annotation of auscultation findings by physicians was avoided. This is a crucial point as machine learning is based on detecting subtle patterns in data. Only when using high‐quality training data, noise that masks these patterns can be sufficiently reduced. Furthermore, the data in our study were pre‐processed before they were used to train the DNN. In this pre‐processing, attributes of the audio data were isolated that have been shown to be essential for pattern recognition in audio files.^27^, ^28^ With this hybrid approach, our DNN showed similar accuracy as board‐certified cardiologists. The precisely defined ground truth in conjunction with the preprocessing of the audio data compensates for a comparatively low patient number.

A potential limitation of our study is that we only included a few patients with moderate aortic stenosis. This is due to the fact that the study was conducted with data from patients admitted to a tertiary teaching hospital for specialized valve therapy. Moreover, patients with only moderate valve disease are challenging to identify because they are often completely asymptomatic and therefore not under medical surveillance. Similarly, the DNN was trained predominantly with normal‐flow, high‐gradient AS. Probably the present algorithm will have worse performance in low‐flow, low‐gradient AS because the flow properties over the valve and thereby the sound characteristics are entirely different. This fact naturally lowers the sensitivity, but this condition affects only a small portion of the patients.

In the present study, a machine learning algorithm was trained to detect patients with aortic stenosis. Patients with other valvular diseases like hypertrophic obstructive cardiomyopathy were not included, and few patients with pure mitral regurgitation participated. Thus, the presented algorithm is far from perfect, and this study can only be the first step in introducing artificial intelligence for valvular heart disease into everyday clinical practice. On the other side, it also shows that artificial intelligence can, in principle, be helpful in the auscultation of heart sounds.

### 4.1 Clinical perspectives

The present study gives proof of concept that AI‐assisted auscultation can provide results on a high expert level concerning the detection of aortic stenosis. Integrating this technology in an electronic stethoscope could be the next step to upgrade this system for everyday clinical use. A stethoscope that indicates a warning in the event of certain valve defects would be conceivable in the foreseeable future. Introducing such a device in countries that cannot provide comprehensive medical care has already been proposed years ago.^29^

## CONFLICTS OF INTEREST

The authors declare no conflicts of interest.

## Data Availability

Data available on request due to privacy/ethical restrictions.
